# RNA virome diversity and *Wolbachia* infection in individual *Drosophila simulans* flies

**DOI:** 10.1099/jgv.0.001639

**Published:** 2021-10-27

**Authors:** Ayda Susana Ortiz-Baez, Mang Shi, Ary A. Hoffmann, Edward C. Holmes

**Affiliations:** ^1^​ Marie Bashir Institute for Infectious Diseases and Biosecurity, School of Life and Environmental Sciences and School of Medical Sciences, The University of Sydney, Sydney, New South Wales 2006, Australia; ^2^​ School of BioSciences, Bio21 Institute, University of Melbourne, Parkville, Victoria 3010, Australia

**Keywords:** evolution, *Drosophila simulans*, meta-transcriptomics, phylogeny, RNA virome, *Wolbachia*

## Abstract

The endosymbiont bacteria of the genus *Wolbachia* are associated with multiple mutualistic effects on insect biology, including nutritional and antiviral properties. Members of the genus *Wolbachia* naturally occur in fly species of the genus *Drosophila*, providing an operational model host for studying how virome composition may be affected by its presence. *Drosophila simulans* populations can carry a variety of strains of members of the genus *Wolbachia *, with the *w*Au strain associated with strong antiviral protection under experimental conditions. We used *D. simulans* sampled from the Perth Hills, Western Australia, to investigate the potential virus protective effect of the *w*Au strain of *Wolbachia* on individual wild-caught flies. Our data revealed no appreciable variation in virus composition and abundance between individuals infected or uninfected with *Wolbachia* associated with the presence or absence of *w*Au. However, it remains unclear whether *w*Au might affect viral infection and host survival by increasing tolerance rather than inducing complete resistance. These data also provide new insights into the natural virome diversity of *D. simulans*. Despite the small number of individuals sampled, we identified a repertoire of RNA viruses, including nora virus, galbut virus, thika virus and La Jolla virus, that have been identified in other species of the genus *Drosophila*. Chaq virus-like sequences associated with galbut virus were also detected. In addition, we identified five novel viruses from the families *Reoviridae*, *Tombusviridae*, *Mitoviridae* and *Bunyaviridae*. Overall, this study highlights the complex interaction between *Wolbachia* and RNA virus infections and provides a baseline description of the natural virome of *D. simulans*.

## Data Summary

The authors confirm all supporting data, code and protocols have been provided within the article or through supplementary data files.

## Introduction

The members of the alpha-proteobacterium genus *Wolbachia* (order *Rickettsiales *) are widespread endosymbionts of arthropods and nematodes (i.e. filarial and plant-parasitic nematodes) that can establish interactions with their hosts ranging from parasitic to mutualistic [1, 2]. The genetic diversity of the genus *Wolbachia* is substantial and currently represented by 11 distinctive supergroups (denoted A–J), although the majority of strains of members of the genus *Wolbachia* belong to supergroups A and B [3] that are estimated to have diverged around 50 million years ago [4]. Although these bacteria are commonly found in reproductive tissues and the germline of their hosts, they have also been found in somatic tissues, such as the brain, salivary glands and gut [5–9], such that understanding infection dynamics in detail is not a trivial matter [7]. Members of the genus *Wolbachia* primarily spread by vertical inheritance through transovarian transmission. However, the presence of members of the genus *Wolbachia* in a diverse range of host species indicates that horizontal transmission, probably through antagonistic interactions (i.e. herbivory, parasitism and predation), also contributes to the dissemination of the bacteria in nature [4, 10].

The occurrence of bacteria of the genus *Wolbachia* in insects is often associated with their ability to manipulate host reproductive mechanisms and induce a range of alterations, including parthenogenesis, feminization, cytoplasmic incompatibility and sex-ratio distortion [11]. Among these, cytoplasmic incompatibility is the most common phenotypic effect, and as such represents an appealing approach for vector population control. In this case, embryonic lethality is contingent on the infection status and the strain type harboured by males and females [2]. In addition, the study of *Wolbachia *–host interactions has revealed a variety of mutualistic effects on host biology [1, 12]. For instance, in filarial nematodes and the parasitoid wasp *Asobara tabida*, the presence of some strains of members of the genus *Wolbachia* has been positively associated with developmental processes, fertility and host viability [12–14]. Furthermore, nutritional mutualism between members of the genus *Wolbachia* and the bedbug *Cimex lectularius* as well as *Wolbachia *-infected planthoppers, has been suggested as a means to explain B vitamin supplementation [15–17].

Arguably the most important outcome of *Wolbachia* infection in insects is its potential for virus-blocking, which also provides a basis for intervention strategies based on the control of arbovirus transmission. This seemingly antiviral effect of members of the genus *Wolbachia* has been well documented in some species of insects, including flies and mosquitoes. A striking example involves the transinfection of *Aedes aegypti* mosquitoes with a strain of the genus *Wolbachia* infecting *Drosophila melanogaster* (*w*Mel). *A. aegypti* is the primary vector of a number of important arboviruses, including members of the species *Dengue virus*, *Zika virus* and *Chikungunya virus*, and the establishment of the *w*Mel strain in wild mosquito populations represents a powerful and promising approach to decrease virus transmission [18, 19]. Although the underlying mechanisms remain to be fully determined, it has been suggested that members of the genus *Wolbachia* can modify the host environment or boost basal immunity to viruses by pre-stimulating the immune response of their hosts [20]. Potential antiviral mechanisms affected by members of the genus *Wolbachia* include gene expression of the Toll pathway, RNA interference, and modification of the host oxidative environment that probably trigger an antiviral immune response and hence limit infection [20–22].

Unlike *A. aegypti* mosquitoes, members of the genus *Wolbachia* naturally occur in species of the genus *Drosophila*, providing a valuable model system to study *Wolbachia *-related virus protection [23, 24]. Natural populations of species of the genus *Drosophila* can carry a diverse array of insect-specific viruses belonging to the families *Picornaviridae*, *Dicistroviridae*, *Bunyaviridae*, *Reoviridae* and *Iflaviridae* amongst others [25]. The co-occurrence of members of the genus *Wolbachia* in *D. melanogaster* has been associated with increased survival and different levels of resistance to laboratory viral infections in fly stocks under experimental conditions [23, 26]. For example, *Wolbachia *-infected flies containing the dicistrovirus Drosophila C virus (DCV) showed a delay in mortality compared with *Wolbachia *-free flies [26]. In contrast, other studies found no or limited effect of members of the genus *Wolbachia* on viral protection, as well as on virus prevalence and abundance in field-collected flies [25, 27]. Such contrasting data emphasize the need for further research efforts to characterize the effect of strains of the genus *Wolbachia* on virus composition in members of the genus *Drosophila* in nature.

Although the origin of *D. simulans* is thought to have been in East Africa or Madagascar, this species now has a cosmopolitan distribution [28]. In Australia, *D. simulans* has been recorded along both the east and west coasts as well as Tasmania, with the earliest record dating to 1956 [29]. Human mobility and human-mediated activities have been associated with the introduction and spread of both *D. simulans* and *Wolbachia* into Australia, where wild fly populations occur near human settlements, feeding and breeding on a variety of horticultural crops [30, 31]. Several strains of members of the genus *Wolbachia* from supergroups A and B can naturally occur in populations of *D. simulans* (e.g. *w*Au, *w*Ri, *w*Ha, *w*Ma and *w*No) [32, 33]. Of these, *w*Au is associated with strong antiviral protection against Flock House virus (FHV) (*Nodaviridae*) and DCV (*Dicistroviridae*) under experimental conditions [32]. The *w*Au infection in Australia was one of the first infections by members of the genus *Wolbachia* identified as showing no cytoplasmic incompatibility, despite being widespread at a low to intermediate frequency [34]. *w*Au increased in frequency along the east coast of Australia until it was replaced by *w*Ri that exhibits cytoplasmic incompatibility. However, unlike *w*Au, *w*Ri has not yet reached the Australian west coast [30]. In this study, we used a meta-transcriptomic (i.e. RNA shotgun sequencing) approach to determine the virome diversity of individual field-collected *D. simulans* flies from Western Australia, and investigated how this virome diversity might be affected by the presence of the *w*Au strain of *Wolbachia *.

## Methods

### *D. simulans* collection and taxonomic identification

Flies used for the virus work performed here were collected at Raeburn Orchards in the Perth Hills in Western Australia (latitude −32.1036°, longitude 116.0695°), in July 2018 using banana bait. The frequency of members of the genus *Wolbachia* at two other locations in the area (Roleystone, latitude −32.1396°, longitude 116.0701°; Cannington, latitude −32.0243°, longitude 115.9363°) was also established with additional samples. Taxonomic identification to the species level was conducted on the basis of the morphology of reproductive traits of males and via DNA barcoding (*cox1* gene marker). Field-collected flies were maintained at 19 °C under standard laboratory conditions until F1 offspring were raised. Parental and F1 generations were then stored at −80 °C until molecular processing.

### *Wolbachia* detection

*Wolbachia* infection of field females was determined using F1 offspring from each field female. Note that *w*Au is transmitted at 100 % from field females to the F1 laboratory generation [34]. DNA extraction from heads was performed using Chelex 100 Resin (Bio-Rad Laboratories,) [35] as adapted y Shi *et al*. [27]. Screening of natural infection with members of the genus *Wolbachia* was conducted using a real-time PCR/ high-resolution melt assay (RT/HRM) and strain-specific primers targeting a 340 bp region of the surface protein of Wolbachia (*wsp*) gene for *w*Ri and *w*Au strains. The assay was run following the protocol of Kriesner *et al*. [30]. In addition, reads were mapped to reference *Wolbachia wsp* gene sequences for *w*Ri (CP001391.1) and *w*Au (LK055284.1) with BBMap v.37.98 (minid=0.95) (available at https://sourceforge.net/projects/bbmap/).

### RNA extraction and meta-transcriptome sequencing

We screened a total of 16 individual flies to assess the effect of infection with members of the genus *Wolbachia* on virome composition in *D. simulans*. Specimens were rinsed three times in RNA and DNA-free PBS solution (GIBCO). Total RNA from individual flies was extracted using the RNeasy Plus Mini Kit (Qiagen) following the manufacturer’s instructions. RNA-seq libraries were constructed using a TruSeq total RNA Library Preparation Kit (Illumina). Host ribosomal depletion was performed using a Ribo-Zero Gold rRNA Removal Kit (Human/Mouse/Rat) (Illumina) and paired-end transcriptome sequencing was performed on the HiSeq2500 platform (Illumina). Libraries from *Wolbachia *-negative and -positive infected flies were run in two separate lanes.

### *De novo* meta-transcriptome assembly and viral genome annotation

The overall quality assessment of reads was conducted in FastQC and Trimmomatic [36]. A *de novo* assembly of RNA-Seq data was performed using MEGAHIT v.1.1.3, with default parameters [37]. Assembled contigs were then annotated through comparisons against the NCBI nonredundant (NCBI-nr) database using DIAMOND v2.0.4 [38], with a cut-off e-value <1e^−05^. To identify protein-encoding sequences, open reading frames (ORFs) were predicted in positive and reverse-complement strands, with a minimum length of 600 nt between two stop codons using the GetOrf program (EMBOSS) [39]. Functional annotation was carried out using InterProScan v5.39–77.0 [40], and the HMMer software (http://hmmer.org/) was used to perform sequence-profile searches against the Pfam HMM database. To expand the *de novo* assembled contigs of known viruses, the reads were mapped against reference genomic sequences. Provisional virus names were derived from geographic locations in the Perth Hills, Western Australia.

### Estimates of viral abundance

Viral abundance was assessed using the number of reads per million (RPM). This metric quantifies the number of reads per million mapped to a given contig assembly over the total number of reads. RPM values lower than 0.1 % of the highest count for each virus across samples were presumed to be index-hopping artefacts and excluded from the remaining analyses [41]. To compare abundance levels, reads were mapped to reference ribosomal and mitochondrial genes from *Wolbachia* (*16S* and *cox1*) and *D. simulans* (*rpl32* and *cox1*), as well as against all the RNA viruses identified in the annotation analyses. Mapping was performed using BBMap v.37.98 (minid=0.95) (available at https://sourceforge.net/projects/bbmap/).

### Sequence alignment and phylogenetic analysis

RNA viral sequences identified in *D. simulans* were compared with homologous reference sequences retrieved from the NCBI GenBank database and aligned with MAFF v7.450 (E-INS-I algorithm) [42]. Phylogenetic trees for these data were then inferred using sequences of the conserved RNA-dependent RNA polymerase (RdRp) gene. To this end, both the best-fit model of amino acid substitution and phylogenetic relationships were estimated using the maximum likelihood (ML) [43] approach implemented in IQ-TREE v1.6.12 [44]. Nodal support was estimated combining the SH-like approximate likelihood ratio test (SH-aLRT) and the Ultrafast Bootstrap Approximation (UFboot) [45]. Redundant contigs with over 99 % amino acid similarity were excluded. For those libraries containing viruses that were unlikely to be associated with *Drosophila*, taxonomic profiling and read mapping to components of the fly microbiome and diet were conducted using the CCMetagen software (default settings) [46] and BBMap v.37.98 (minid=0.95).

### Statistical analysis

The assumption of data normality was assessed by visual inspection and using Kolmogorov–Smirnov (K-S) and Shapiro–Wilk’s tests. As the data was not normally distributed, a Mann–Whitney–Wilcoxon test was used to compare the RNA virome composition with respect to the presence/absence of members of the genus *Wolbachia *. Comparisons were made using raw and log-transformed data corresponding to RPM values (i.e. viral abundance) for each library. All analyses were performed using R software package rstatix (available at https://rpkgs.datanovia.com/rstatix/).

## Results

A total of 272 female flies were wild-caught in the Perth Hills, Western Australia and tested for *Wolbachia* infection through their F1s. The overall prevalence of members of the genus *Wolbachia* was 63.6 % (173 out of 272), with frequencies at the three sampled locations varying from 54.8 % (Raeburn Orchard, *n*=73) to 63.8 % (Roleystone, *n*=130) and 72.5 % (Cannington, *n*=69). We randomly selected 16 flies from the Raeburn Orchard field females for individual sequencing and RNA virus screening, representing eight *Wolbachia *-positive and eight *Wolbachia *-negative specimens.

We identified the *Wolbachia* strain in *D. simulans* using sequence-specific primers targeting the *wsp* gene. We further confirmed the occurrence of *Wolbachia* by mapping the reads back to the *w*Ri and *w*Au *wsp* genes. Most of the *Wolbachia *-infected flies showed a median coverage >100 reads, number of mapping reads >40, and coverage percentage >90 % to the reference *w*Au strain, confirming that infected flies harbour *w*Au rather than *w*Ri. No reads mapped to the *wsp* gene for library RAPP88 (Table S1, available in the online version of this article) despite the positive infection status determined using a *Wolbachia *-specific qPCR assay.

**Fig. 1.**
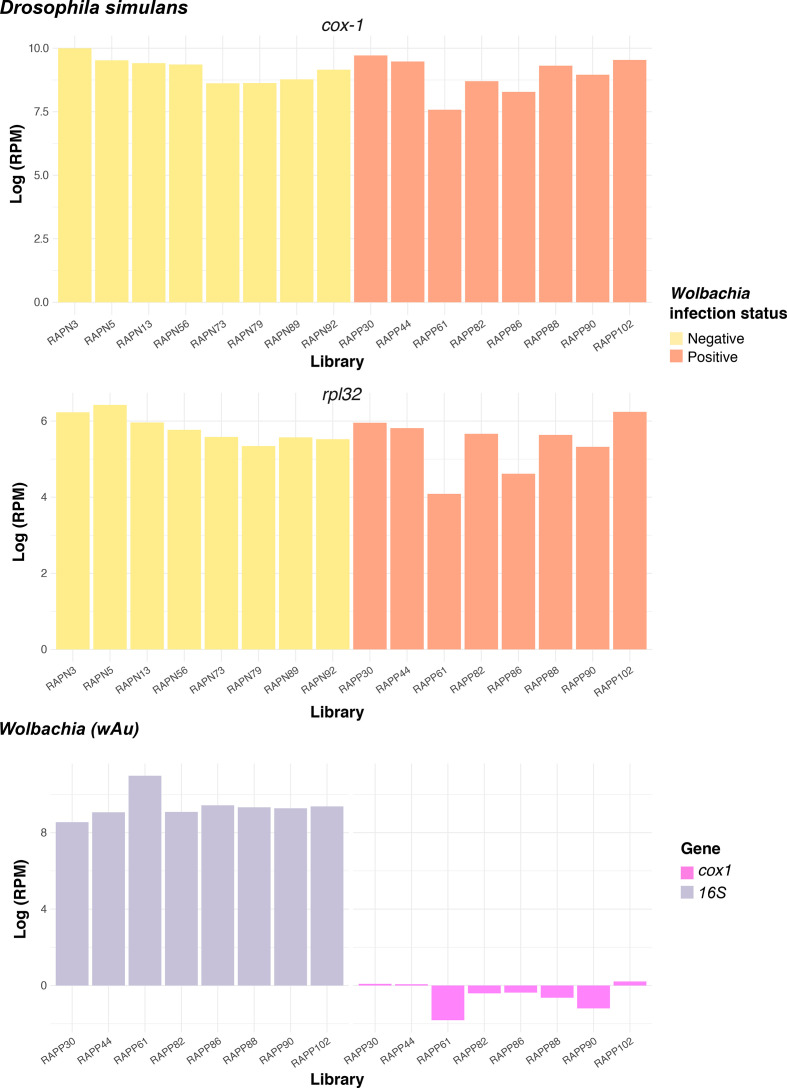
Comparison of the abundance levels of reference genes in *Wolbachia *-positive and *Wolbachia *-negative individual *D. simulans* (*rpl32* and *cox-1*) and species of the genus *Wolbachia* (*16S* and *cox1*).

For comparison of virus diversity among libraries we mapped the reads of each library to stably expressed genes: *16S* and *cox1* in *Wolbachia* and *rpl32* and *cox1* in *D. simulans*. This provided an internal control to identify any effect on viral abundance due to potential biases introduced during RNA extraction or library preparation. Although, as expected, there was moderate variation in the abundance values, expression levels of reference maker genes were relatively stable across libraries in both *Wolbachia* and *D. simulans* (Fig. 1).

**Fig. 2.**
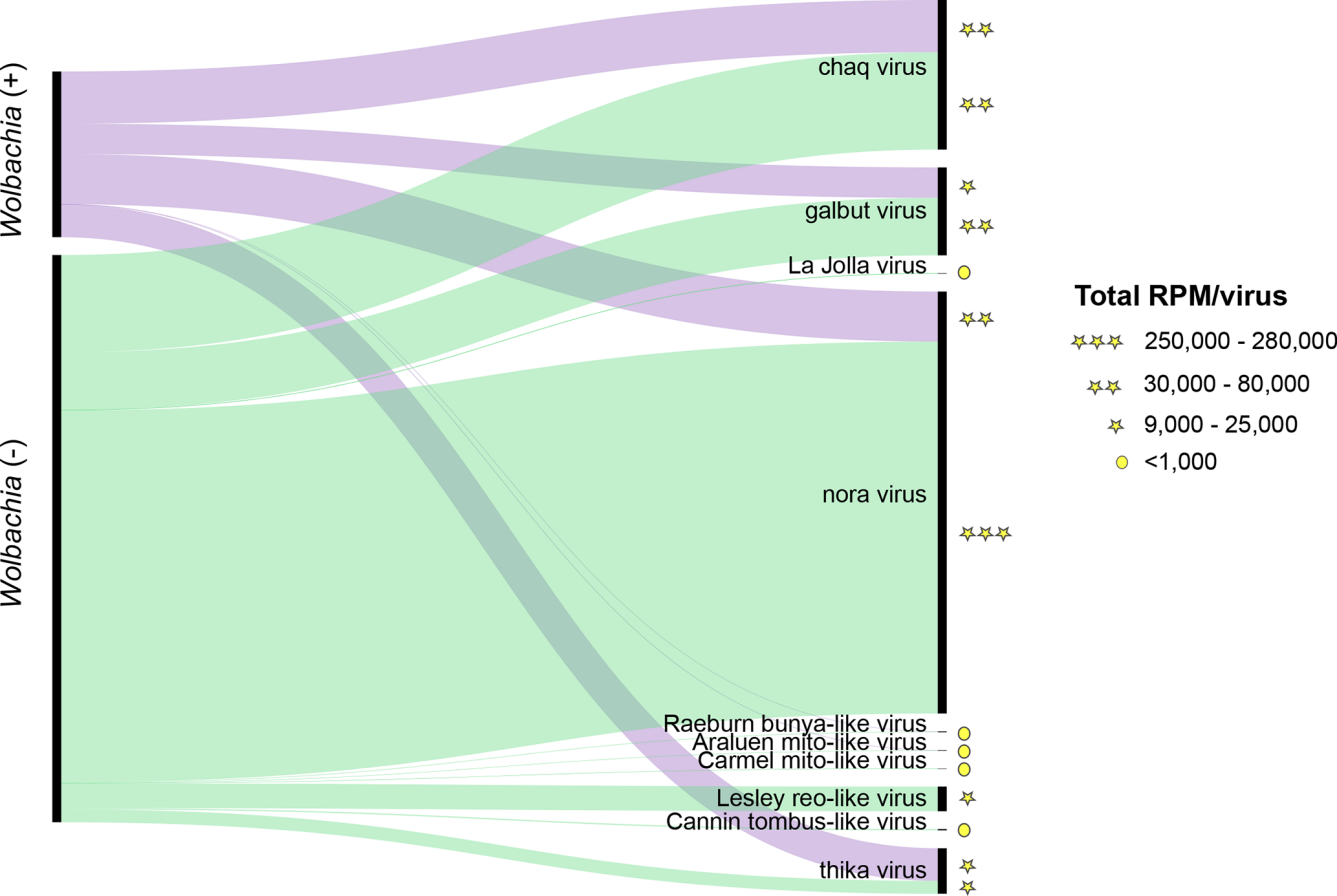
Comparison of viruses found in *Wolbachia *-positive and *Wolbachia *-negative *D. simulans*. The thickness of links is proportional to the total abundance (RPM) of each virus across the samples studied. The range of RPM values are represented with stars and circles.

**Fig. 3.**
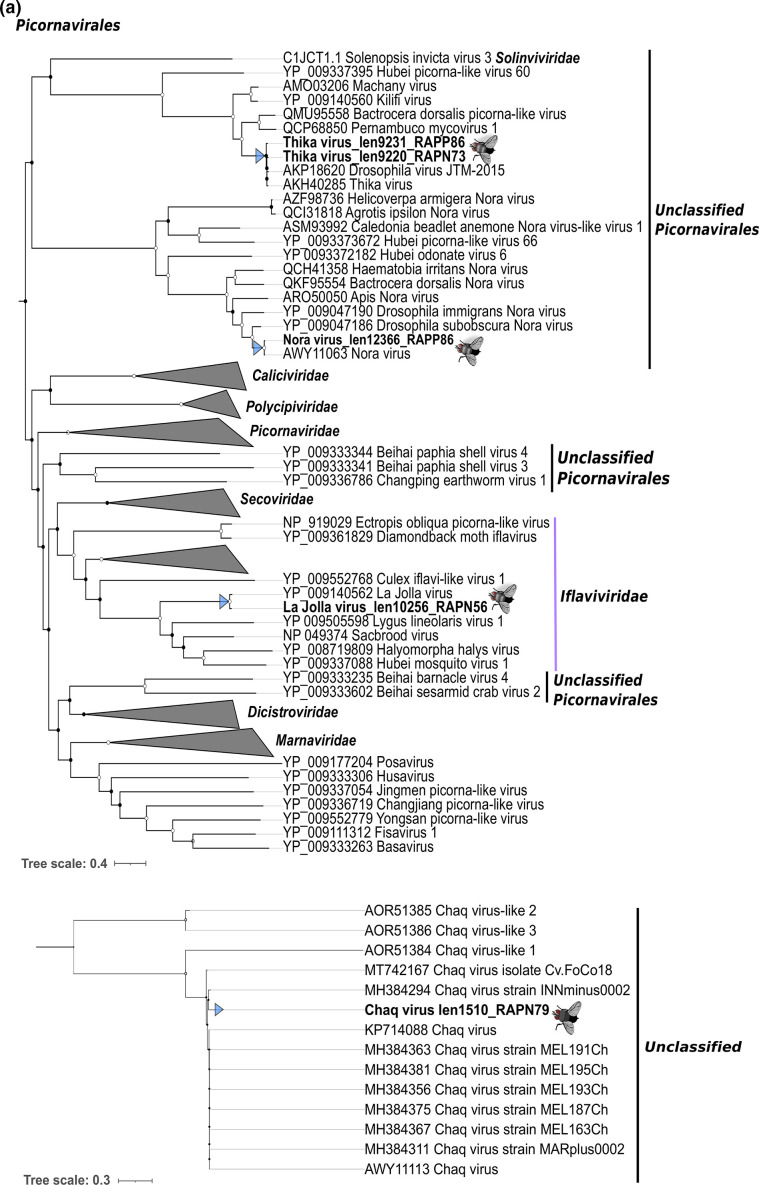

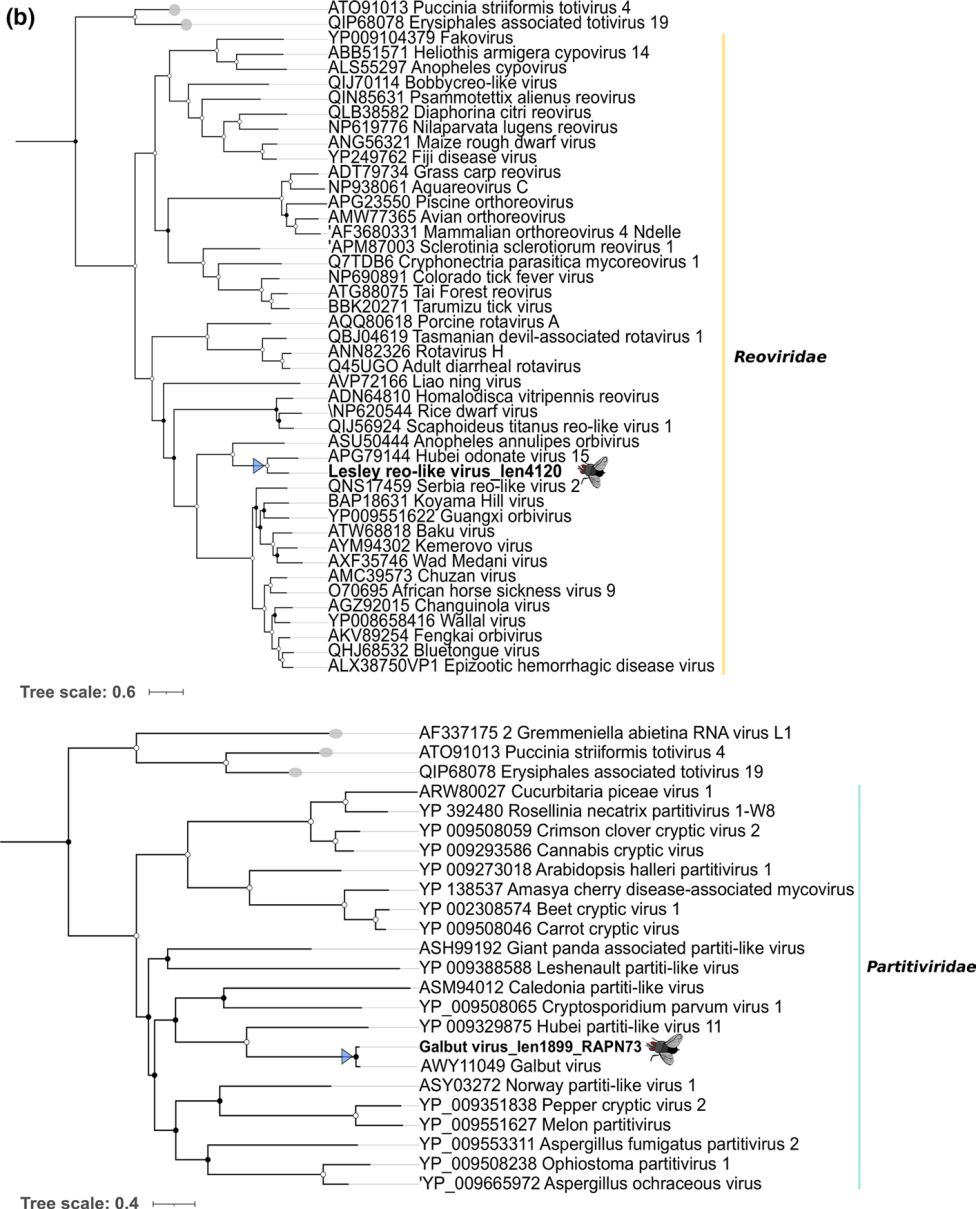

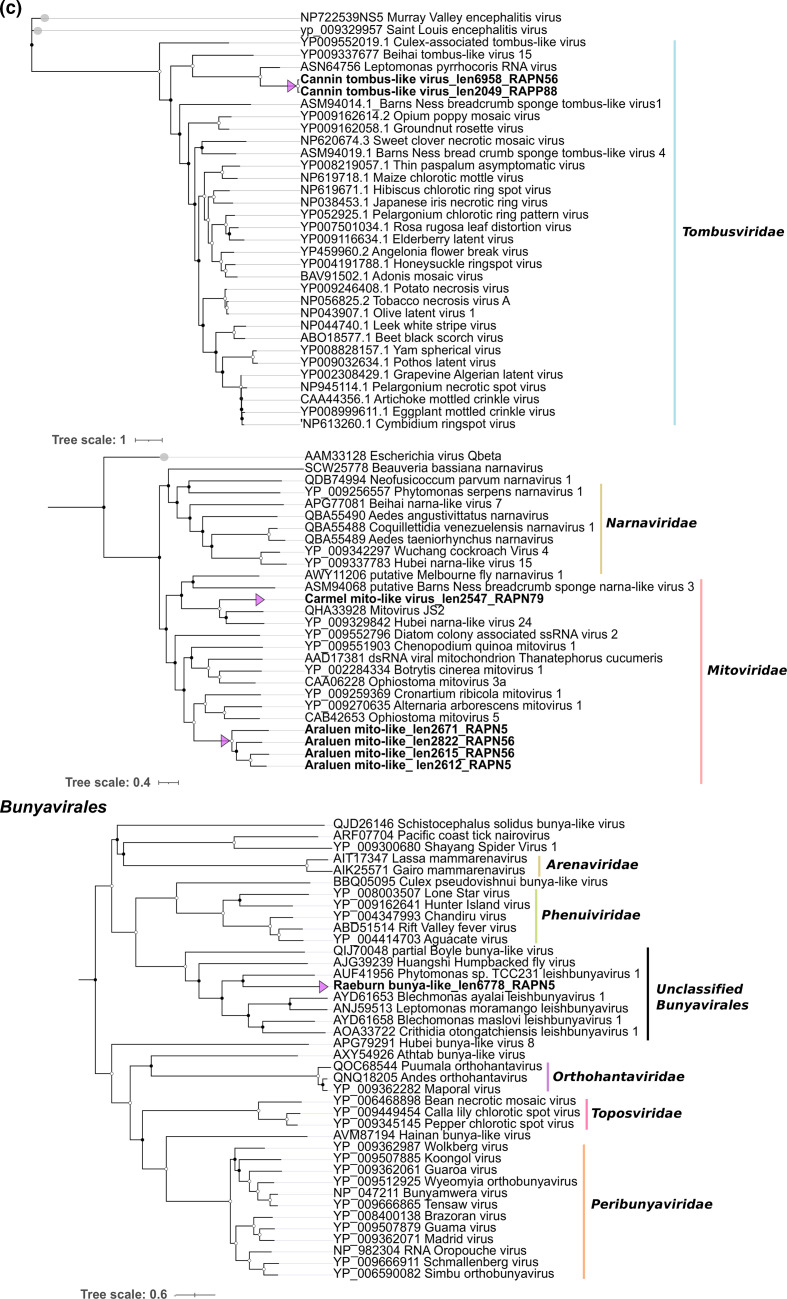
Maximum likelihood phylogenetic trees of the viruses and virus-like sequences identified from *D. simulans*. The phylogenies were inferred based on the amino acid sequences of the RdRp of six virus taxonomic groups, whereas for chaq virus-like sequences we used a protein of unknown function. Virus family trees were rooted with relevant outgroups that are indicated with grey tips. Order-level trees and the chaq virus phylogeny (for which no suitable outgroup existed) were midpoint rooted. Coloured arrow tips represent probable (**a, b**) *Drosophila*-associated viruses and (**c**) non-*Drosophila*-associated viruses (i.e. those that were more probably associated with a component of fly diet or microbiome). Nodal support values greater than 80 % (SH-aLRT) and 95 % (UFboot) are indicated with white circular shapes at the nodes. Branch lengths are projected using scale bars below each tree.

**Fig. 4.**
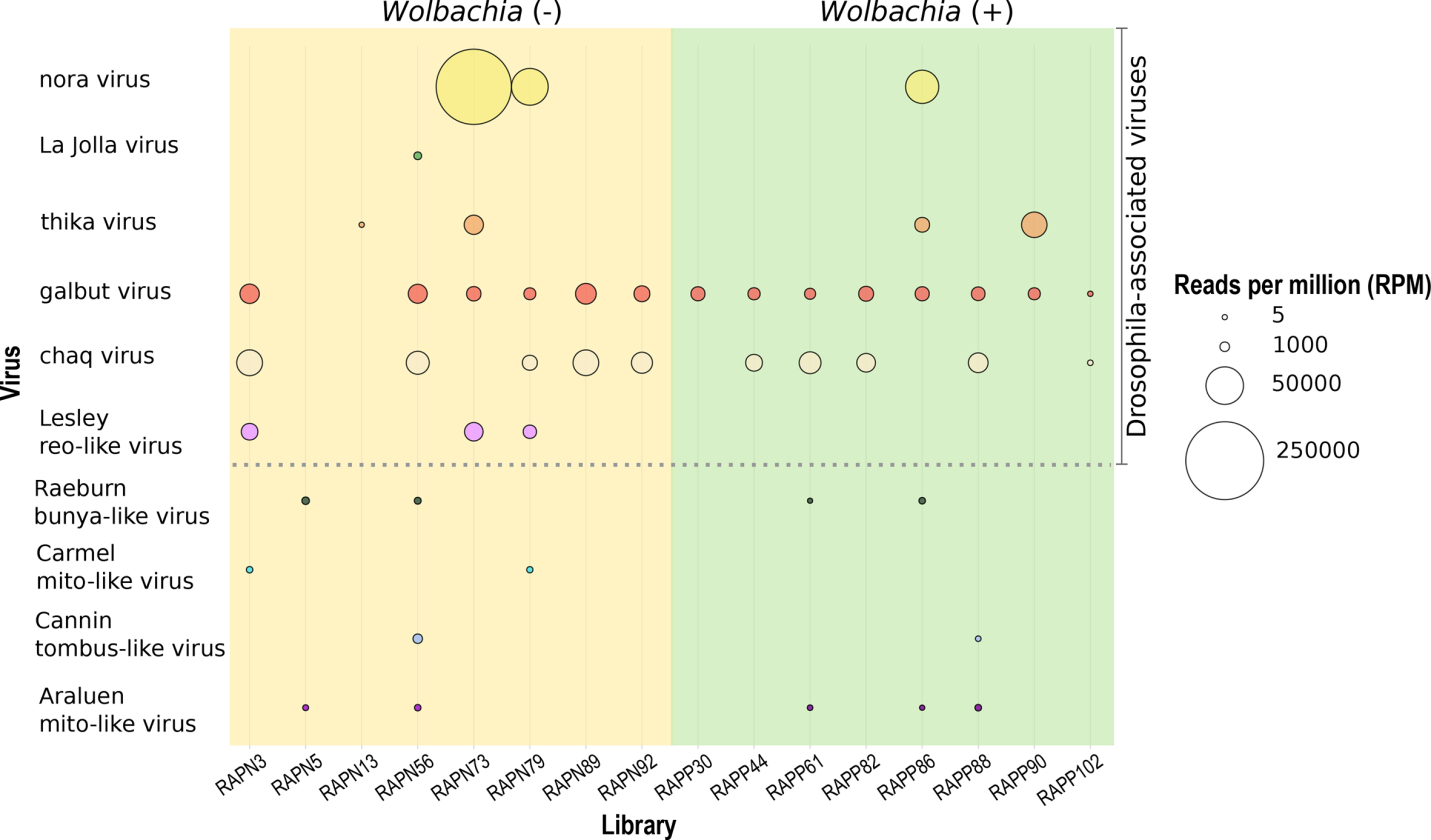
Representation of virome composition and abundance (RPM) across *Wolbachia *-positive and -negative libraries. Each library represents an individual *D. simulans* fly. All reads likely to be due to index-hopping have been excluded.

Overall, we detected nine viruses in the 16 individual *D. simulans* studied here, five of which were novel (Fig. 2). Specifically, four viruses shared high sequence identity at the amino acid level (>96 %, e-value=0.00E^+00^–4.2E^−41^) to the RdRp of known RNA viruses, whereas the newly discovered viruses shared only between 32.6 and 62.6% amino acid identity to the best viral hit (e-value=0.00E^+00^–1.4E^−06^) (Tables 1, and S4). Similarly, phylogenetic analysis of the known virus sequences identified revealed close relationships with known *Drosophila*-associated viruses: galbut virus (*Partitiviridae*), La Jolla virus (*Iflaviridae*), thika virus (*Picornaviridae*) and nora virus (*Picornaviridae*) (Fig. 3). In addition, we identified contigs related to ‘chaq virus-like’ sequences (>85 % amino acid sequence similarity). The novel viruses identified, that did not share close phylogenetic relationships to known viruses, were: Raeburn bunya-like virus (*Bunyaviridae*), Araluen mito-like virus (*Mitoviridae*), Carmel mito-like virus (*Mitoviridae*), Lesley reo-like virus (*Reoviridae*), and Cannin tombus-like virus (*Tombusviridae*) (Fig. 3). Similarity searches against the NCBI/nr database revealed that individual flies carried multiple invertebrate-associated viruses from different virus families. For example, up to six viruses were observed in a single *w*Au-negative library (RAPN56) (Fig. 4, Table S2).

**Table 1.** Summary of sequence similarity searches for viruses against the NCBI non-redundant database. Viral sequences listed below correspond to those included in phylogenetic analyses

Query sequence	Library	* Wolbachia* infection	Length (nt)	Best match against the NCBI/nr database	Similarity	e-value
k119_3301_len12366_nora virus	RAPP86	+	12 366	AWY11063.1 putative replicase [nora virus]	98.7	0.00E^+00^
k119_19486_len10256_La Jolla virus	RAPN56	−	10 256	AWY11061.1 putative polyprotein [La Jolla virus]	98	0.00E^+00^
k119_20553_len9231_thika virus	RAPP86	+	9231	YP_009140561.1 putative polyprotein [thika virus]	96.2	0.00E^+00^
k119_5914_len9220_thika virus	RAPN73	−	9220	YP_009140561.1 putative polyprotein [thika virus]	97.1	0.00E^+00^
k119_3227_len6958_Cannin tombus-like virus	RAPN56	−	6958	ASN64756.1 putative RNA-dependent RNA polymerase, partial [*Leptomonas pyrrhocoris* RNA virus]	44.6	1.80E^−96^
k119_2329_len2049_Cannin tombus-like virus	RAPP88	+	2049	ASN64759.1 putative RNA-dependent RNA polymerase, partial [*Leptomonas pyrrhocoris* RNA virus]	48.4	3.80E^−95^
k119_4103_len1899_galbut virus	RAPN73	−	1899	AWY11176.1 putative RNA-dependent RNA polymerase [galbut virus]	96.7	0.00E^+00^
k119_13353_len1510_chaq virus	RAPN79	−	1510	AWY11113.1 hypothetical protein [chaq virus]	85.9	1.6E^−153^
k119_2075_len4120_ Lesley reo-like virus	RAPN73	−	4120	APG79144.1 RNA-dependent RNA polymerase [Hubei odonate virus 15]	48.6	0.00E^+00^
k119_10165_len2547_Carmel mito-like virus	RAPN79	−	2547	YP_009329842.1 RNA-dependent RNA polymerase [Hubei narna-like virus 24]	32.7	2.0E^−76^
k119_273_len2671_Araluen mito-like virus	RAPN5	−	2671	QDH87474.1 RNA-dependent RNA polymerase, partial [*Mitovirus* sp.]	40.3	8.0E^−96^
k119_22084_len2612_Araluen mito-like virus	RAPN5	−	2612	QDH87474.1 RNA-dependent RNA polymerase, partial [*Mitovirus* sp.]	43.2	2.3E^−103^
k119_14037_len2615_Araluen mito-like virus	RAPN56	−	2615	QDH87474.1 RNA-dependent RNA polymerase, partial [*Mitovirus* sp.]	41.7	1.7E^−98^
k119_14318_len2822_Araluen mito-like virus	RAPN56	−	2822	QDH87474.1 RNA-dependent RNA polymerase, partial [*Mitovirus* sp.]	38.1	9.7E^−92^

Some of the newly discovered RNA viruses identified here were probably infecting hosts other than *D. simulans*, and hence might be associated with the fly diet or microbiome. Specifically, these viruses were closely related to *Phytomonas* sp. TCC231 leishbunyavirus 1 (in the case of Raeburn bunya-like virus), *Leptomonas pyrrhocoris* RNA virus (Cannin tombus-like virus) and two mito-like viruses (Araluen mito-like virus and Carmel mito-like virus) (Fig. 3, Table S3), that are associated with trypanosomatid protozoans and fungal hosts, respectively. In addition, taxonomic composition analyses as well as read mapping to common components of *Drosophila* diet or microbiome revealed that 0.1 and 0.05 % of all non-rRNA reads mapped to fungi and trypanosomatids (*cox1* gene marker), respectively. Hence, multiple microorganisms were present within individual fly libraries which may explain the occurrence of viruses not directly associated with *Drosophila* (files available at https://doi.org/10.6084/m9.figshare.c.5466690). In contrast, Lesley reo-like virus is likely to be a *bona fide* arthropod virus since it grouped with viruses previously detected in odonates and mosquitoes. In addition, it exhibited only approximately 24 % nucleotide similarity with those reoviruses previously reported to be contaminants in *Drosophila* cell culture [25]. This indicates that Lesley reo-like virus is not a component of known contaminants and more is likely to be part of the natural *D. simulans* virome. The five newly identified viruses in this study corresponded to full or nearly complete genomes (see below). However, for the majority of the known viruses of *Drosophila* we only were able to identify ORFs encoding the RdRp: the exceptions were La Jolla virus and thika virus, for which we also predicted structural components corresponding to coat and capsid proteins.

We next characterized the virome profile present in *D. simulans* in relation to the *w*Au infection status (Fig. 2, Tables 1 and S4). Accordingly, we identified a slightly higher number (*n*=9) of viruses in *Wolbachia *-negative flies compared with *Wolbachia *-positive flies (*n*=6). Among these, galbut virus, nora virus, thika virus, as well as three novel viruses identified in this study – Raeburn bunya-like virus, Araluen mito-like virus and Cannin tombus-like virus – were present in *D. simulans* regardless of *Wolbachia* infection. Likewise, ‘chaq virus-like’ sequences were observed co-occurring with galbut virus in the two groups of *D. simulans*. In contrast, La Jolla virus, as well as the novel Carmel mito-like virus and Lesley reo-like virus, were only found in *w*Au-negative flies. Overall, assembled viral contigs displayed high sequence similarity at the nucleotide and amino acid level within and between libraries and regardless of the presence or absence of members of the genus *Wolbachia* (Table S3).

**Fig. 5.**
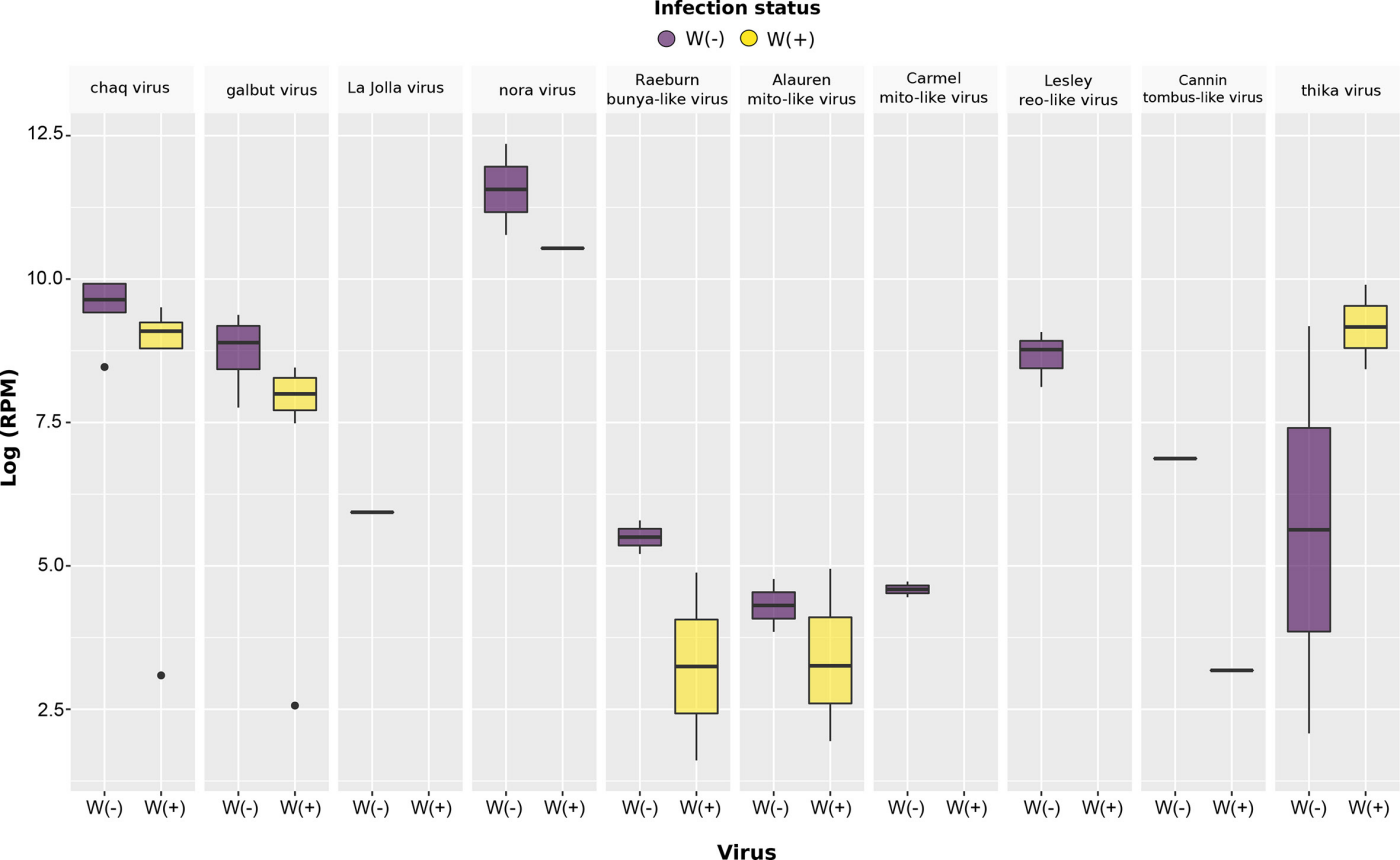
Abundance distribution of nine RNA viruses and the chaq virus-like sequences identified across individual *Wolbachia *-positive and *Wolbachia-*negative *D. simulans*. A non-significant difference was observed between *Wolbachia *-infected and uninfected flies using the Mann-Whitney U test.

We also assessed the potential effect of infection with members of the genus *Wolbachia* on the abundance of RNA viruses present in *w*Au-infected and *w*Au-uninfected flies. Overall, the number of non-rRNA reads represented approximately 50 % of the total number of reads (*n*=743 389 696 pair-end reads) (Fig. S1). Furthermore, the RPM values among viruses infecting *Wolbachia *-negative and -positive infected flies was highly heterogeneous, ranging from 47 to 232 346 and 5 to 37 688 virus RPM, respectively. With the exception of thika virus, viruses present in both *w*Au-positive and *w*Au-negative flies were 1.87–40.17-fold more abundant in the *w*Au-negative individuals than *w*Au-positive *D. simulans*. In contrast, the abundance of thika virus was 0.39-fold higher in the *Wolbachia *-positive flies (Fig. 3, Table S2). However, despite this variation in virus abundance levels between groups, there was a non-significant difference between *w*Au-negative and *w*Au-positive *D. simulans* (Mann–Whitney–Wilcoxon test; Fig. 5). In the case of the viruses only detected in the *w*Au-negative flies, La Jolla virus was present in a single library in moderate abundance (RPM=378), whilst the newly discovered Lesley reo-like virus was detected in four out of eight libraries (RPM=3360–8749) (Table S2). Although an interesting result, the limited sample size (*n*=16) means that these observations should be taken with caution and that larger sample sizes are needed for corroboration.

## Discussion

The occurrence and spread of *Wolbachia* infection has been widely documented in natural populations of *Drosophila* [10, 30, 47]. Indeed, *D. simulans* is commonly used as an experimental model to investigate the interactions within the tripartite *Drosophila–Wolbachia*–virus system. In Australia, *D. simulans* can be naturally infected with two strains of *Wolbachia* from supergroup A *w*Au and *w*Ri. While *w*Ri has been gradually displacing *w*Au in eastern Australia, reflected in the changing infection frequencies in surveyed populations since 2004, *D. simulans* from the west coast of Australia only harbour *w*Au [30]. A simple and plausible explanation for this difference is the geographic separation of *D. simulans* populations inhabiting the east and west coasts of Australia and the challenging environmental conditions posed by the intervening desert [30].

We corroborated the presence of *Wolbachia* infection across samples by identifying the *wsp*, *16S* and *cox1* marker genes. The lack of reads mapping to the library RAPP88 might reflect either low levels of *wsp* RNA molecules present in the input for library preparation or high variability compared with the reference sequence. Although *Wolbachia* density was not experimentally assessed, the similar levels of *16S* and *cox-1* abundance across libraries indicate no appreciable biases in the library preparation and RNA sequencing steps.

Estimates from previous surveys indicated that the frequency of the *w*Au strain in Western Australia exceeded 50 % in *D. simulans* [30]. This is consistent with the data provided here and indicates that *Wolbachia* might be present in a significant proportion of the natural fly population, at least around Perth. Although *w*Au does not cause cytoplasmic incompatibility, its spread is suggested to confer fitness advantages (increased survival and/or reproduction) to the host, including antiviral protection [48, 49], that might favour its spread and prevent the bacteria from being eliminated from *D. simulans* populations [30, 50]. However, our comparison of *Wolbachia *-infected and -uninfected *D. simulans* in western Australia revealed no clear effect of *Wolbachia* infection on virome composition and viral abundance between *Wolbachia* infected and uninfected animals. Although our analysis is based on a small sample of individual flies, the apparent absence of a *Wolbachia *-mediated virus protection effect in natural *D. simulans* is compatible with previous findings on *D. melanogaster* naturally infected with *w*Mel in eastern Australia [27], in which virus protection was not observed regardless of the *Wolbachia* infection status and *Wolbachia* density. Even so, the absence of a significant association between *w*Au infection and virus diversity does not necessarily translate into a homogeneous effect of *w*Au on the different viruses identified here. For example, it is plausible that the restricted presence of La Jolla virus and the newly identified Lesley reo-like virus in *Wolbachia *-free flies could reflect some effect of antiviral protection in *D. simulans* [27, 51]. Indeed, contrasting results were observed in *D. melanogaster*, where La Jolla virus was widely distributed across different libraries [27]. Although this might provide insights into *w*Au-virus interactions, studies based on larger sample sizes are clearly needed to determine whether the apparent association between La Jolla virus and *Wolbachia *-uninfected flies observed here is an artefact due to small sample sizes. Indeed, it is notable that La Jolla virus was so rarely detected in the *D. simulans* flies studied here.

It has previously been shown that the *w*Au strain of *Wolbachia* has a protective role against virus infection in *D. simulans* when flies are challenged with FHV and DCV in a laboratory setting [24, 32]. Moreover, the *w*Au strain is protective against the dengue (DENV) and zika (ZIKV) viruses in *Aedes aegypti* mosquitoes [52]. Although our observation of an apparent lack of *Wolbachia *-mediated antiviral protection contrasts with those obtained previously, it is likely that differences may depend on *Wolbachia *–host species combinations and natural or artificial viral infections, which may also explain the contrasting results for La Jolla virus. Indeed, most of the available studies have documented the antiviral effect in transinfected insect hosts with non-natural strains of *Wolbachia* and viruses under laboratory conditions, as opposed to the study of the natural virome undertaken here.

It is noteworthy that ecological variables such as temperature might affect *Wolbachia *–virus–host interactions. Here, we collected flies during the Western Australian winter (mean temperature of 21 °C daylight time) and the specimens were maintained at 19 °C under laboratory conditions. Lower temperatures have been associated with an increase in viral resistance against DCV in *D. melanogaster* infected with *w*Mel and *w*MelCS [53]. Similarly, variations in host developmental temperature have been associated with differences in *Wolbachia *-mediated virus blocking in natural populations [53]. In this context, flies developed at lower temperature (18 °C) exhibited a reduction in *Wolbachia *-conferred antiviral protection. On the other hand, the presence of *Wolbachia* has been suggested to influence host temperature preferences. For instance, *w*Ri and *w*Ho strains seem to manipulate *D. simulans* flies to seek cooler temperatures [54]. Although the effect of temperature on *w*Au and *D. simulans* need to be tested, this indicates that the results observed here as well as a protective scenario might be temperature-dependent. This highlights the importance of careful future studies of the interactions within the host–virus*–Wolbachia* system along with environmental factors in natural populations [55–57].

As well as the small sample size, an important caveat of our work is that we explored the *Wolbachia *-mediated virus protection in terms of virus abundance levels reflected in RPM values. This provides insights into virus resistance, but not into tolerance or host survival. Thus, it is still possible that *Wolbachia* is increasing tolerance to virus infection as has been documented for DCV [32]. In addition, although we were not able to assess *Wolbachia* density, results of previous studies have indicated that *w*Au is maintained at high-density in *D. simulans* and has a role in virus blocking [58]. Further research is clearly needed to assess these features in natural populations to determine any link with antiviral protection.

Collectively, comparisons of the virome composition in *w*Au infected and uninfected *D. simulans* revealed the presence of natural and relatively highly abundant *Drosophila*-associated viruses in both groups [25, 27, 59]. Consistently with results from previous studies, we noted the co-occurrence of chaq virus-like sequences and galbut virus, supporting the idea that chaq virus might be part of a satellite–helper virus system or an additional segment associated with galbut virus [27, 60]. In addition to insect-associated viruses, we identified viruses that are likely to infect other hosts and hence were likely to be associated with components of the diet or microbiome of *D. simulans* [61]. For instance, novel viruses from the families *Tombusviridae* and *Bunyaviridae* were related to viruses in trypanosomatid protozoa (*Leptomonas* and *Leishmania*). Similarly, given their normal host range distribution, the novel viruses from the family *Narnaviridae* might be associated with fungal hosts. Evidence of trypanosomatids and fungi in the gut of several species of *Drosophila,* with effects on larvae eclosion and pupation times [61, 62], has been reported. This, in turn, highlights the extent to which Australian *D. simulans* can be parasitized in nature [62–66].

In sum, we provide a preliminary framework for assessing the effect of the *w*Au strain on the virome of *D. simulans*, using a meta-transcriptomic analysis of individual *w*Au-infected and uninfected flies. In doing so we identified *Drosophila*-associated viruses along with five novel viruses likely to be associated with fly diet or microbiome. Although our sample size is small, we saw no detectable *Wolbachia *-associated antiviral effect on virus composition and abundance, although the approach taken prevented us from drawing conclusions on virus tolerance. Further research employing larger sample sizes over broad spatial scales, including different *Wolbachia–Drosophila* combinations, will enable a more nuanced understanding of *Wolbachi* a–virus dynamics in wild *Drosophila* populations.

## Supplementary Data

Supplementary material 1

Supplementary material 2

## References

[1] Zug R, Hammerstein P (2015). Bad guys turned nice? A critical assessment of *Wolbachia* mutualisms in arthropod hosts. Biol Rev Camb Philos Soc.

[2] Ross PA, Turelli M, Hoffmann AA (2019). Evolutionary ecology of *Wolbachia* releases for disease control. Annu Rev Genet.

[3] Ros VID, Fleming VM, Feil EJ, Breeuwer JAJ (2009). How diverse is the genus *Wolbachia*? Multiple-gene sequencing reveals a putatively new *Wolbachia* supergroup recovered from spider mites (Acari: Tetranychidae. Appl Environ Microbiol.

[4] Scholz M, Albanese D, Tuohy K, Donati C, Segata N (2020). Large scale genome reconstructions illuminate Wolbachia evolution. Nat Commun.

[5] Zouache K, Voronin D, Tran-Van V, Mousson L, Failloux A-B (2009). Persistent *Wolbachia* and cultivable bacteria infection in the reproductive and somatic tissues of the mosquito vector *Aedes albopictus*. PLoS One.

[6] Dobson SL, Bourtzis K, Braig HR, Jones BF, Zhou W (1999). *Wolbachia* infections are distributed throughout insect somatic and germ line tissues. Insect Biochem Mol Biol.

[7] Frydman HM, Li JM, Robson DN, Wieschaus E (2006). Somatic stem cell niche tropism in *Wolbachia*. Nature.

[8] Tsai K-H, Lien J-C, Huang C-G, Wu W-J, Chen W-J (2004). Molecular (sub) grouping of endosymbiont *Wolbachia* infection among mosquitoes of Taiwan. J Med Entomol.

[9] Strunov A, Kiseleva E (2016). *Drosophila melanogaster* brain invasion: Pathogenic *Wolbachia* in central nervous system of the fly. Insect Sci.

[10] Turelli M, Cooper BS, Richardson KM, Ginsberg PS, Peckenpaugh B (2018). Rapid global spread of *w*Ri-like *Wolbachia* across multiple *Drosophila*. Curr Biol.

[11] O’Neill SL, Hoffmann A, Werren J (1997). Influential Passengers: Inherited Microorganisms and Arthropod Reproduction.

[12] Iturbe-Ormaetxe I (2007). Current Opinion in Microbiology.

[13] Dedeine F, Vavre F, Fleury F, Loppin B, Hochberg ME (2001). Removing symbiotic *Wolbachia* bacteria specifically inhibits oogenesis in a parasitic wasp. Proc Natl Acad Sci U S A.

[14] Hoerauf A, Nissen-Pähle K, Schmetz C, Henkle-Dührsen K, Blaxter ML (1999). Tetracycline therapy targets intracellular bacteria in the filarial nematode *Litomosoides sigmodontis* and results in filarial infertility. J Clin Invest.

[15] Nikoh N, Hosokawa T, Moriyama M, Oshima K, Hattori M (2014). Evolutionary origin of insect–*Wolbachia* nutritional mutualism. Proc Natl Acad Sci U S A.

[16] Ju J-F, Bing X-L, Zhao D-S, Guo Y, Xi Z (2020). *Wolbachia* supplement biotin and riboflavin to enhance reproduction in planthoppers. ISME J.

[17] Hosokawa T, Koga R, Kikuchi Y, Meng XY, Fukatsu T (2010). *Wolbachia* as a bacteriocyte-associated nutritional mutualist. Proc Natl Acad Sci U S A.

[18] Tantowijoyo W, Andari B, Arguni E, Budiwati N, Nurhayati I (2020). Stable establishment of wMel *Wolbachia* in *Aedes aegypti* populations in Yogyakarta, Indonesia. Barker CM, editor. PLoS Negl Trop Dis.

[19] Moreira LA, Iturbe-Ormaetxe I, Jeffery JA, Lu G, Pyke AT (2009). A *Wolbachia* symbiont in *Aedes aegypti* limits infection with Dengue, Chikungunya, and Plasmodium. Cell.

[20] Rancès E, Ye YH, Woolfit M, McGraw EA (2012). The relative importance of innate immune priming in *Wolbachia*-mediated dengue interference. PLoS Pathog.

[21] Zug R, Hammerstein P (2015). *Wolbachia* and the insect immune system: what reactive oxygen species can tell us about the mechanisms of *Wolbachia*–host interactions. Front Microbiol.

[22] Terradas G, Joubert DA, McGraw EA (2017). The RNAi pathway plays a small part in *Wolbachia*-mediated blocking of dengue virus in mosquito cells. Sci Rep.

[23] Teixeira L, Ferreira A, Ashburner M (2008). The bacterial symbiont *Wolbachia* induces resistance to RNA viral infections in *Drosophila melanogaster*. PLoS Biol.

[24] Martinez J, Longdon B, Bauer S, Chan Y-S, Miller WJ (2014). Symbionts commonly provide broad spectrum resistance to viruses in insects: a comparative analysis of *Wolbachia* strains. PLoS Pathog.

[25] Webster CL, Waldron FM, Robertson S, Crowson D, Ferrari G (2015). The discovery, distribution, and evolution of viruses associated with *Drosophila melanogaster*. PLoS Biol.

[26] Hedges LM, Brownlie JC, O’Neill SL, Johnson KN (2008). *Wolbachia* and virus protection in insects. Science.

[27] Shi M, White VL, Schlub T, Eden J-S, Hoffmann AA (2018). No detectable effect of *Wolbachia w*Mel on the prevalence and abundance of the RNA virome of *Drosophila melanogaster*. Proc Biol Sci.

[28] Lachaise D, Cariou M-L, David JR, Lemeunier F, Tsacas L (1988). Evolutionary Biology.

[29] Mather W (1960). Additions to the *Drosophila* fauna of Australia. Univ Queensl Pap.

[30] Kriesner P, Hoffmann AA, Lee SF, Turelli M, Weeks AR (2013). Rapid sequential spread of two *Wolbachia* variants in *Drosophila simulans*. Charlat S, editor. PLoS Pathog.

[31] Parsons PA, Bock IR (1979). The population biology of Australian *Drosophila*. Annu Rev Ecol Syst.

[32] Osborne SE, Leong YS, O’Neill SL, Johnson KN (2009). Variation in antiviral protection mediated by different *Wolbachia* strains in *Drosophila simulans*. Schneider DS, editor. PLoS Pathog.

[33] Casiraghi M, Bordenstein SR, Baldo L, Lo N, Beninati T (2005). Phylogeny of *Wolbachia pipientis* based on *gltA, groEL* and *ftsZ* gene sequences: Clustering of arthropod and nematode symbionts in the F supergroup, and evidence for further diversity in the *Wolbachia* tree. Microbiology (Reading).

[34] Hoffmann AA, Clancy D, Duncan J (1996). Naturally-occurring *Wolbachia* infection in *Drosophila simulans* that does not cause cytoplasmic incompatibility. Heredity (Edinb).

[35] Endersby NM, Mckechnie SW, Vogel H, Gahan LJ, Baxter SW (2005). Microsatellites isolated from diamondback moth, *Plutella xylostella* (L.), for studies of dispersal in Australian populations. Mol Ecol Notes.

[36] Bolger AM, Lohse M, Usadel B (2014). Trimmomatic: a flexible trimmer for Illumina sequence data. Bioinformatics.

[37] Grabherr MG, Haas BJ, Yassour M, Levin JZ, Thompson DA (2011). Full-length transcriptome assembly from RNA-Seq data without a reference genome. Nat Biotechnol.

[38] Buchfink B, Xie C, Huson DH (2015). Fast and sensitive protein alignment using DIAMOND. Nat Methods.

[39] Rice P, Longden L, Bleasby A (2000). EMBOSS: The European Molecular Biology Open Software Suite. Trends Genet.

[40] Zdobnov EM, Apweiler R (2001). InterProScan - an integration platform for the signature-recognition methods in InterPro. Bioinformatics.

[41] Lay CL, Shi M, Buček A, Bourguignon T, Lo N (2020). Unmapped RNA virus diversity in termites and their symbionts. Viruses.

[42] Katoh K, Standley DM (2013). MAFFT Multiple Sequence Alignment Software Version 7: Improvements in Performance and Usability. Mol Biol Evol.

[43] Felsenstein J (1981). Evolutionary trees from DNA sequences: A maximum likelihood approach. J Mol Evol.

[44] Nguyen L-T, Schmidt HA, von Haeseler A, Minh BQ (2015). IQ-TREE: A fast and effective stochastic algorithm for estimating maximum-likelihood phylogenies. Mol Biol Evol.

[45] Hoang DT, Chernomor O, Von Haeseler A, Minh BQ, Vinh LS (2018). UFBoot2: Improving the ultrafast bootstrap approximation. Mol Biol Evol.

[46] Marcelino VR, Clausen P, Buchmann JP, Wille M, Iredell JR (2019). CCMetagen: comprehensive and accurate identification of eukaryotes and prokaryotes in metagenomic data. bioRxiv.

[47] Turelli M, Hoffmann AA (1991). Rapid spread of an inherited incompatibility factor in California *Drosophila*. Nature.

[48] Ogunlade ST, Adekunle AI, Meehan MT, Rojas DP, McBryde ES (2020). Modeling the potential of *w*Au-*Wolbachia* strain invasion in mosquitoes to control *Aedes*-borne arboviral infections. Sci Rep.

[49] Mancini MV, Herd CS, Ant TH, Murdochy SM, Sinkins SP (2020). *Wolbachia* strain *w*Au efficiently blocks arbovirus transmission in *Aedes albopictus*. Samy AM, editor. PLoS Negl Trop Dis.

[50] Correa CC, Ballard JWO (2016). Wolbachia associations with insects: Winning or losing against a master manipulator. Front Ecol Evol.

[51] Habayeb MS, Cantera R, Casanova G, Ekström J-O, Albright S (2009). The *Drosophila* Nora virus is an enteric virus, transmitted via feces. J Invertebr Pathol.

[52] Ant TH, Herd CS, Geoghegan V, Hoffmann AA, Sinkins SP (2018). The *Wolbachia* strain *w*Au provides highly efficient virus transmission blocking in *Aedes aegypti*. Schneider DS, editor. PLOS Pathog.

[53] Chrostek E, Martins N, Marialva MS, Teixeira L (2020). *Wolbachia*-conferred antiviral protection is determined by developmental temperature. bioRxiv.

[54] Hague MTJ, Caldwell CN, Cooper BS (2020). Pervasive effects of *Wolbachia* on host temperature preference. MBio.

[55] Johnson KN (2015). The impact of *Wolbachia* on virus infection in mosquitoes. Viruses.

[56] Cao L-J, Jiang W, Hoffmann AA (2019). Life history effects linked to an advantage for wAu *Wolbachia* in *Drosophila*. Insects.

[57] Fenton A, Johnson KN, Brownlie JC, Hurst GDD (2011). Solving the *Wolbachia* paradox: Modeling the tripartite interaction between host, *Wolbachia*, and a natural enemy. Am Nat.

[58] Osborne SE, Iturbe-Ormaetxe I, Brownlie JC, O’Neill SL, Johnson KN (2012). Antiviral protection and the importance of *Wolbachia* density and tissue tropism in *Drosophila simulans*. Appl Environ Microbiol.

[59] Palmer WH, Medd NC, Beard PM, Obbard DJ (2018). Isolation of a natural DNA virus of *Drosophila melanogaster*, and characterisation of host resistance and immune responses. PLoS Pathog.

[60] Cross ST, Maertens BL, Dunham TJ, Rodgers CP, Brehm AL (2020). Partitiviruses infecting *Drosophila melanogaster* and *Aedes aegypti* exhibit efficient biparental vertical transmission. J Virol.

[61] Ebbert MA, Marlowe JL, Burkholder JJ (2003). Protozoan and intracellular fungal gut endosymbionts in *Drosophila*: prevalence and fitness effects of single and dual infections. J Invertebr Pathol.

[62] Wilfert L, Longdon B, Ferreira AGA, Bayer F, Jiggins FM (2011). Trypanosomatids are common and diverse parasites of *Drosophila*. Parasitology.

[63] Chandler JA, James PM (2013). Discovery of trypanosomatid parasites in globally distributed *Drosophila* species. Braga ÉM, editor. PLoS One.

[64] Ebbert MA, Burkholder JJ, Marlowe JL (2001). Trypanosomatid prevalence and host habitat choice in woodland *Drosophila*. J Invertebr Pathol.

[65] Lemaitre B, Nicolas E, Michaut L, Reichhart JM, Hoffmann JA (1996). The dorsoventral regulatory gene cassette *spatzle*/*Toll*/*Cactus* controls the potent antifungal response in Drosophila adults. Cell.

[66] Naranjo-Lázaro JM, Mellín-Rosas MA, González-Padilla VD, Sánchez-González JA, Moreno-Carrillo G (2014). Susceptibility of *Drosophila suzukii* Matsumura (Diptera: Drosophilidae) to entomophatogenic fungi. Southwestern Entomologist.

