# Supplementary material for: RNA virome diversity and Wolbachia infection in individual Drosophila simulans flies
Source: J Gen Virol. 2021 Oct 27;102(10):001639. doi: 10.1099/jgv.0.001639 (PMC8604192; doi:10.1099/jgv.0.001639)
Supplement: Supplementary material 1 [file jgv-102-1639-s001.pdf]

Figure S1. Ribosomal and non-ribosomal reads in each sequencing library. (A) Number of ribosomal/non-ribosomal reads within the total number of reads. (B) Percentage of ribosomal/non-ribosomal reads within the total number of reads.

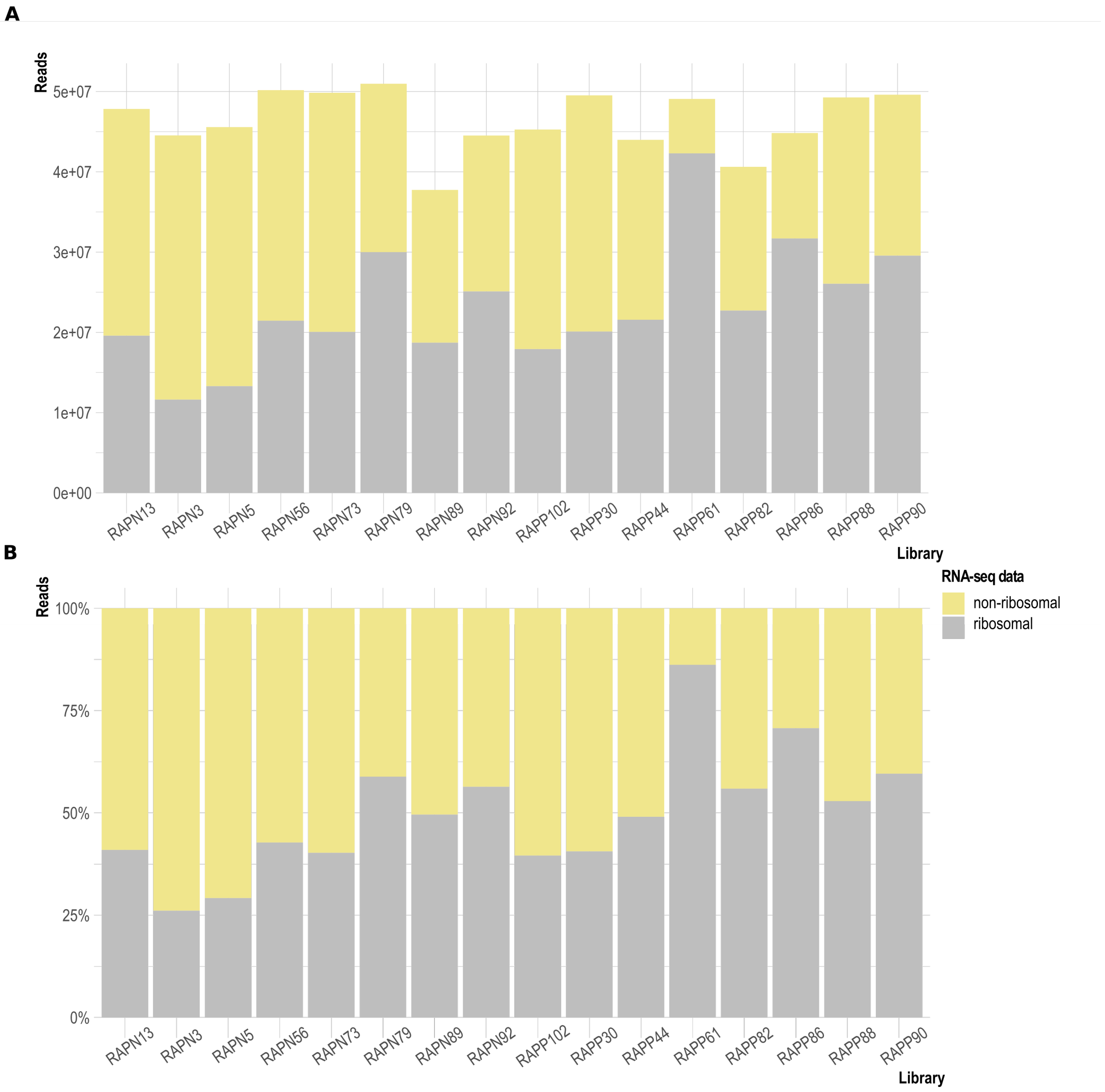

Table S2. Abundance values for individual viruses and virus-like sequences identified in this study across *Wolbachia* infected/uninfected *D. simulans*. The abundance values for a given virus correspond to the number of reads per million mapped reads (RPM). Viruses with RPM values lower than 0.1% of the highest abundance for each virus are shown in grey and assumed to represent index-hopping (and were excluded from additional analyses).

	Wolbachia-negative *D. simulans*							
	**RAPN13**	**RAPN3**	**RAPN5**	**RAPN56**	**RAPN73**	**RAPN79**	**RAPN89**	**RAPN92**
**nora virus (Picorna-like)**	8*	5*	5*	7*	232346	47585	5*	4*
**La Jolla virus (Picorna-like)**	0	0	0	378	0	0	0	0
**thika virus (Picorna-like)**	8*	10*	14*	6*	9694	6*	7*	5*
**galbut virus (Partiti-like)**	1*	9865	0	9370	4264	2346	11799	5650
**chaq virus (Partiti-like)**	0	20577	0	15380	0	4756	20293	12303
**Lesley reo-like virus**	0	6438	0	0	8749	3361	1*	0
**Cannin tombus-like virus**	0	0	0	964	0	0	0	0
**Raeburn bunya-like virus**	0	0	328	183	0	0	0	0
**Carmel mito-like virus**	0	113	0	0	0	86	0	0
**Araluen mito-like virus**	0	0	47	118	0	0	0	0
	Wolbachia-positive *D. simulans*							
	**RAPP102**	**RAPP30**	**RAPP44**	**RAPP61**	**RAPP82**	**RAPP86**	**RAPP88**	**RAPP90**
**nora virus (Picorna-like)**	1*	1*	1*	0	1*	37688	1*	1*
**La Jolla virus (Picorna-like)**	0	0	0	0	0	0	0	0
**thika virus (Picorna-like)**	8*	8*	7*	2*	7*	4580	6*	19940
**galbut virus (Partiti-like)**	13	3920	2463	1784	4708	3980	3597	2412
**chaq virus (Partiti-like)**	22	0	6578	13454	8881	15*	10343	0
**Lesley reo-like virus**	0	0	0	0	0	0	0	0
**Cannin tombus-like virus**	0	0	0	0	0	0	24	0
**Raeburn bunya-like virus**	0	0	0	5	0	132	0	0
**Carmel mito-like virus**	0	0	0	0	0	0	0	0
**Araluen mito-like virus**	0	0	0	26	0	7	141	0

* RPM values excluded as they likely correspond to index-hopping artefacts during RNA-sequencing.

Table S3. List of virus contigs selected for phylogenetic analysis based on levels of sequence similarity.

Virus	Nº of contigs (Libraries)	% nt identity	% aa identity	Representative sequence (library)*
nora virus (Picorna-like)	4 (RAPN73, RAPN79, RAPP86)	99.21 - 100	99.65 - 100	k119_3301_len12366_nora_virus (RAPP86)
La Jolla virus (Picorna-like)	1 (RAPN56)	n/a	n/a	k119_19486_len10256_La_Jolla_virus (RAPN56)
thika virus (Picorna-like)	3 (RAPN73, RAPP86, RAPP90)	91.90-99.99	96.15 - 99.97	k119_20553_len9231_thika_virus (RAPP86), k119_5914_len9220_thika_virus (RAPN73)
Cannin tombus-like virus	3 (RAPN56, RAPP88)	94.34 - 99.27	87.44 - 99.41	k119_2329_len2049_cannin tombus-like virus (RAPP88), k119_3227_len6958_cannin tombus-like virus (RAPN56)
galbut virus (Partiti-like)	14 (RAPP30, RAPP44, RAPP61, RAPP82, RAPP86, RAPP88, RAPP90, RAPP102, RAPN73)	96.75-100	98.71-100	k119_4103_len1899_galbut_virus (RAPN73)
chaq virus (Unclassified)	12 (RAPN3, RAPN56, RAPN56, RAPN79, RAPN89, RAPN92 RAPP102, RAPP44 RAPP61, RAPP82 RAPP86, RAPP88)	84.48-100	98.49-99.69	k119_13353_len1510_chaq virus (RAPN79)
Carmel mito-like virus	2 (RAPN3, RAPN79)	98.63	99.27	k119_10165 _len2547_carmel mito-like virus (RAPN79)
Araluen mito-like virus	RAPP61, RAPP86, RAPP88, RAPN5, RAPN56	39.44-100	33.37-98.57	k119_14037_len2615_araluen mito-like virus (RAPN56), k119_22084_len2612_araluen mito-like virus (RAPN5), k119_14318 _len2822_araluen mito-like virus (RAPN56), k119_273_len2671_araluen mito-like virus (RAPN5)
Lesley reo-like virus	3 (RAPN3, RAPN73, RAPN79)	99.55 - 99.83	88.62 - 99.85	k119_2075_len4120_lesley reo-like virus (RAPN73)
Raeburn bunya-like virus	3 (RAPN5, RAPN56, RAPP86)	99.40-99.56	99.40-99.54	k119_6166_len6778_raeburn bunya-like virus (RAPN5)

Table S4. Summary of sequence similarity searches for the total of virus contigs against the NCBI non-redundant database.

Query sequence	Library	*Wolbachia* infection	Length (nt)	Best match against the BLAST/nr database	Similarity	e-value
k119_18666_len12382_nora virus	RAPN79	-	12382	AWY11063.1 putative replicase [Nora virus]	98.7	0.00E+00
k119_2699_len5328_nora virus	RAPN73	-	5328	AKH67631.1 replication polyprotein [Nora virus]	98.9	0.00E+00
k119_3301_len12366_nora virus	RAPP86	+	12366	AWY11063.1 putative replicase [Nora virus]	98.7	0.00E+00
k119_7728_len7167_nora virus	RAPN73	-	7167	AWY11063.1 putative replicase [Nora virus]	98.6	0.00E+00
k119_19486_len10256_La Jolla virus	RAPN56	-	10256	AWY11061.1 putative polyprotein [La Jolla virus]	98	0.00E+00
k119_5105_len9362_thika virus	RAPP90	+	9362	YP_009140561.1 putative polyprotein [Thika virus]	96.2	0.00E+00
k119_20553_len9231_thika virus	RAPP86	+	9231	YP_009140561.1 putative polyprotein [Thika virus]	96.2	0.00E+00
k119_5914_len9220_thika virus	RAPN73	-	9220	YP_009140561.1 putative polyprotein [Thika virus]	97.1	0.00E+00
k119_6595_len6974_Cannin tombus-like virus	RAPN56	-	6974	ASN64759.1 putative RNA-dependent RNA polymerase, partial [Leptomonas pyrrhocoris RNA virus]	48.4	1.30E-94
k119_3227_len6958_Cannin tombus-like virus	RAPN56	-	6958	ASN64756.1 putative RNA-dependent RNA polymerase, partial [Leptomonas pyrrhocoris RNA virus]	44.6	1.80E-96
k119_2329_len2049_Cannin tombus-like virus	RAPP88	+	2049	ASN64759.1 putative RNA-dependent RNA polymerase, partial [Leptomonas pyrrhocoris RNA virus]	48.4	3.80E-95
k119_14665_len1835_galbut virus	RAPP30	+	1835	AWY11176.1 putative RNA-dependent RNA polymerase [Galbut virus]	97	0.00E+00
k119_7000_len1801_galbut virus	RAPN79	-	1801	AWY11176.1 putative RNA-dependent RNA polymerase [Galbut virus]	96.1	0.00E+00
k119_15592_len2183_galbut virus	RAPN56	-	2183	AWY11176.1 putative RNA-dependent RNA polymerase [Galbut virus]	96.1	0.00E+00
k119_17720_len1823_galbut virus	RAPP88	+	1823	AWY11176.1 putative RNA-dependent RNA polymerase [Galbut virus]	96.1	0.00E+00
k119_4103_len1899_galbut virus	RAPN73	-	1899	AWY11176.1 putative RNA-dependent RNA polymerase [Galbut virus]	96.7	0.00E+00

k119_17804_len1790_galbut virus	RAPN89	-	1790	AWY11176.1 putative RNA-dependent RNA polymerase [Galbut virus]	96.1	0.00E+00
k119_21494_len1682_galbut virus	RAPP86	+	1682	AWY11176.1 putative RNA-dependent RNA polymerase [Galbut virus]	96.9	0.00E+00
k119_18395_len1758_galbut virus	RAPP82	+	1758	AWY11176.1 putative RNA-dependent RNA polymerase [Galbut virus]	96.3	0.00E+00
k119_15212_len1735_galbut virus	RAPP61	+	1735	AWY11176.1 putative RNA-dependent RNA polymerase [Galbut virus]	96.1	0.00E+00
k119_16819_len1733_galbut virus	RAPP44	+	1733	AWY11176.1 putative RNA-dependent RNA polymerase [Galbut virus]	95.9	0.00E+00
k119_15935_len1648_galbut virus	RAPP102	+	1648	AWY11176.1 putative RNA-dependent RNA polymerase [Galbut virus]	96.3	0.00E+00
k119_3037_len1672_galbut virus	RAPP90	+	1672	AWY11176.1 putative RNA-dependent RNA polymerase [Galbut virus]	96.1	0.00E+00
k119_2472_len1724_galbut virus	RAPN3	-	1724	AWY11176.1 putative RNA-dependent RNA polymerase [Galbut virus]	96.1	0.00E+00
k119_14538_len1827_galbut virus	RAPN92	-	1827	AWY11176.1 putative RNA-dependent RNA polymerase [Galbut virus]	96.3	0.00E+00
k119_21572_len1547_chaq virus	RAPN3	-	1547	AWY11113.1 hypothetical protein [Chaq virus]	86.2	3.7E-154
k119_1063_len1169_chaq virus	RAPN56	-	1169	AWY11113.1 hypothetical protein [Chaq virus]	87	2.8E-122
k119_6487_len519_chaq virus	RAPN56	-	519	AWY11113.1 hypothetical protein [Chaq virus]	85.7	4.2E-41
k119_13353_len1510_chaq virus	RAPN79	-	1510	AWY11113.1 hypothetical protein [Chaq virus]	85.9	1.6E-153
k119_1562_len1480_chaq virus	RAPN89	-	1480	AWY11113.1 hypothetical protein [Chaq virus]	85.9	5.9E-153
k119_12859_len1545_chaq virus	RAPN92	-	1545	AWY11113.1 hypothetical protein [Chaq virus]	85.9	1.6E-153
k119_15738_len1430_chaq virus	RAPP102	+	1430	AWY11113.1 hypothetical protein [Chaq virus]	85.6	5.7E-153
k119_9647_len1467_chaq virus	RAPP44	+	1467	AWY11113.1 hypothetical protein [Chaq virus]	85.9	9.0E-154
k119_18560_ len1471_chaq virus	RAPP61	+	1471	AWY11113.1 hypothetical protein [Chaq virus]	86.2	3.1E-154
k119_17462_len1474_chaq virus	RAPP82	+	1474	AWY11113.1 hypothetical protein [Chaq virus]	86.2	3.1E-154

k119_4913_ len1396_chaq virus	RAPP86	+	1396	AWY11113.1 hypothetical protein [Chaq virus]	85.6	2.7E-152
k119_2052_len1639_chaq virus	RAPP88	+	1639	AWY11113.1 hypothetical protein [Chaq virus]	85.6	4.7E-151
k119_13097_len4222_Lesley reo-like virus	RAPN79	-	4222	APG79144.1 RNA-dependent RNA polymerase [Hubei odonate virus 15]	48.6	0.00E+00
k119_6812_len4171_ Lesley reo-like virus	RAPN3	-	4171	APG79144.1 RNA-dependent RNA polymerase [Hubei odonate virus 15]	48.6	0.00E+00
k119_2075_len4120_ Lesley reo-like virus	RAPN73	-	4120	APG79144.1 RNA-dependent RNA polymerase [Hubei odonate virus 15]	48.6	0.00E+00
k119_3302_len2571_Carmel mito-like virus	RAPN3	-	2571	YP_009329842.1 RNA-dependent RNA polymerase [Hubei narna-like virus 24]	32.6	3.80E-76
k119_10165_len2547_Carmel mito-like virus	RAPN79	-	2547	YP_009329842.1 RNA-dependent RNA polymerase [Hubei narna-like virus 24]	32.7	2.0e-76
k119_6166_len6778_Raeburn bunya-like virus	RAPN5	-	6778	AUF41956.1 RNA-dependent RNA polymerase [Phytomonas sp. TCC231 leishbunyavirus 1]	33.8	1.50E-225
k119_10640_len6770_Raeburn bunya-like virus	RAPP86	+	6770	AUF41956.1 RNA-dependent RNA polymerase [Phytomonas sp. TCC231 leishbunyavirus 1]	33.6	3.40E-225
k119_13493_len6778_Raeburn bunya-like virus	RAPN56	-	6778	AUF41956.1 RNA-dependent RNA polymerase [Phytomonas sp. TCC231 leishbunyavirus 1]	33.8	1.50E-225
k119_18643_len616_Raeburn bunya-like virus	RAPP61	+	616	ANJ59510.1 putative RNA dependent RNA polymerase [Leptomonas moramango leishbunyavirus]	50.8	5.6E-41
k119_18154_len397_Araluen mito-like virus	RAPP86	+	397	QDH87577.1 RNA-dependent RNA polymerase [Mitovirus sp.]	41	1.3E-17
k119_18803_len660_Araluen mito-like virus	RAPP86	+	660	QDH87474.1 RNA-dependent RNA polymerase, partial [Mitovirus sp.]	56.6	5.1E-56
k119_2431_len529_Araluen mito-like virus	RAPP61	+	529	QDH87474.1 RNA-dependent RNA polymerase, partial [Mitovirus sp.]	65.5	4.2E-13
k119_4889_len444_Araluen mito-like virus	RAPP61	+	444	QDH89956.1 RNA-dependent RNA polymerase, partial [Mitovirus sp.]	62.6	6.3E-34
k119_7925_len640_Araluen mito-like virus	RAPP61	+	640	QDH87474.1 RNA-dependent RNA polymerase, partial [Mitovirus sp.]	50.5	7.9E-46
k119_11923_len1133_Araluen mito-like virus	RAPP61	+	1133	QDH87474.1 RNA-dependent RNA polymerase, partial [Mitovirus sp.]	37.7	1.7E-35

k119_910_len407_Araluen mito-like virus	RAPP88	+	407	QDH89786.1 RNA-dependent RNA polymerase [Mitovirus sp.]	43	6.8E-11
k119_1774_len2695_Araluen mito-like virus	RAPP88	+	2695	QDH87474.1 RNA-dependent RNA polymerase, partial [Mitovirus sp.]	40.1	1.2E-95
k119_4605_len1802_Araluen mito-like virus	RAPP88	+	1802	QDH87474.1 RNA-dependent RNA polymerase, partial [Mitovirus sp.]	43.3	1.9E-68
k119_14272_len692_Araluen mito-like virus	RAPP88	+	692	QDH86541.1 RNA-dependent RNA polymerase, partial [Mitovirus sp.]	43	1.4E-11
k119_18473_len904_Araluen mito-like virus	RAPP88	+	904	QDH87474.1 RNA-dependent RNA polymerase, partial [Mitovirus sp.]	47.1	2.4E-64
k119_19278_len2543_Araluen mito-like virus	RAPP88	+	2543	QDH87474.1 RNA-dependent RNA polymerase, partial [Mitovirus sp.]	43.2	1.9E-103
k119_19907_len1133_Araluen mito-like virus	RAPP88	+	1133	QDH87474.1 RNA-dependent RNA polymerase, partial [Mitovirus sp.]	35.5	1.4E-29
k119_273_len2671_Araluen mito-like virus	RAPN5	-	2671	QDH87474.1 RNA-dependent RNA polymerase, partial [Mitovirus sp.]	40.3	8.0E-96
k119_2554_len759_Araluen mito-like virus	RAPN5	-	759	QDH86541.1 RNA-dependent RNA polymerase, partial [Mitovirus sp.]	46.7	8.0E-09
k119_2894_len1507_Araluen mito-like virus	RAPN5	-	1507	QDH87474.1 RNA-dependent RNA polymerase, partial [Mitovirus sp.]	33.3	4.4E-43
k119_5165_len1639_Araluen mito-like virus	RAPN5	-	1639	QDH87474.1 RNA-dependent RNA polymerase, partial [Mitovirus sp.]	41.15	4.2E-79
k119_7808_len326_Araluen mito-like virus	RAPN5	-	326	QDH89786.1 RNA-dependent RNA polymerase [Mitovirus sp.]	42.9	2.4E-10
k119_9428_len371_Araluen mito-like virus	RAPN5	-	371	QDH86541.1 RNA-dependent RNA polymerase, partial [Mitovirus sp.]	38.9	1.4E-06
k119_12924_len1504_Araluen mito-like virus	RAPN5	-	1504	QDH87474.1 RNA-dependent RNA polymerase, partial [Mitovirus sp.]	46.9	8.0E-53
k119_22084_len2612_Araluen mito-like virus	RAPN5	-	2612	QDH87474.1 RNA-dependent RNA polymerase, partial [Mitovirus sp.]	43.2	2.3E-103
k119_495 _len2643_Araluen mito-like virus	RAPN56	-	2643	QDH87474.1 RNA-dependent RNA polymerase, partial [Mitovirus sp.]	43.4	2.7E-104
k119_1684_len2692_Araluen mito-like virus	RAPN56	-	2692	QDH87474.1 RNA-dependent RNA polymerase, partial [Mitovirus sp.]	40	8.1E-96
k119_14037_len2615_Araluen mito-like virus	RAPN56	-	2615	QDH87474.1 RNA-dependent RNA polymerase, partial [Mitovirus sp.]	41.7	1.7E-98
k119_14318_len2822_Araluen mito-like virus	RAPN56	-	2822	QDH87474.1 RNA-dependent RNA polymerase, partial [Mitovirus sp.]	38.1	9.7E-92
